# Screening of Different Pea ( *Pisum sativum* L.) Genotypes for Osmotic Stress Resistance Using Selective in Vitro Systems

**DOI:** 10.1002/pei3.70215

**Published:** 2026-09-29

**Authors:** Litun Ahmed Labib, Muhseena Nazneen Mohima, Swagata Ahmed, Md. Zahir Rayhan, Md. Fakhrul Hasan

**Affiliations:** ^1^ Department of Horticulture Patuakhali Science and Technology University Patuakhali Bangladesh; ^2^ Department of Genetics and Plant Breeding Patuakhali Science and Technology University Patuakhali Bangladesh; ^3^ Department of Horticulture EXIM Bank Agricultural University Chapainawabganj Bangladesh; ^4^ Department of Horticulture Gazipur Agricultural University Gazipur Bangladesh

**Keywords:** in vitro screening, osmotic stress, pea ( *Pisum sativum* L.), sorbitol‐induced stress, stress tolerance

## Abstract

Osmotic stress severely limits the growth and yield of pea ( *Pisum sativum* L.). It is essential to identify the osmotic stress‐tolerant genotypes in early stages of growth for optimizing pea production under adverse conditions. This study aimed to assess osmotic stress tolerance in a diverse collection of various pea genotypes using in vitro screening with sorbitol‐induced osmotic stress. The genotypes were cultured on Murashige and Skoog (MS) medium supplemented with three sorbitol concentrations—0.0 M (control), 0.1 M, and 0.2 M. Various key growth parameters were evaluated. Results demonstrated that osmotic stress negatively impacted germination and growth in susceptible varieties, while tolerant varieties exhibited resilience. Among the tested genotypes, BD4142 exhibited the best performance under maximum stress level (0.2 M sorbitol), with a germination rate of 43.33%, notable shoot (19.33 mm) and root (32 mm) lengths, and the highest plant fresh weight (258.32 mg). Further cluster analysis classified the genotypes into four distinct groups: highly susceptible, low osmotic tolerance, moderate osmotic tolerance, and high osmotic tolerance. BD4142 and BD4209 were identified as highly osmotic‐tolerant, where BD4142 emerged as the most stable genotype based on the Multi‐Trait Selection Index (MTSI). These findings indicate that BD4142 could be a promising genotype for cultivation under osmotic stress conditions.

## 1 Introduction

Pea ( *Pisum sativum* L.) is an important leguminous crop with significant agronomic advantages and a wide range of uses in food and feed. Peas are valued for their nutritional profile, which includes high levels of soluble and insoluble fibers, amino acids (notably tryptophan and lysine), complex carbohydrates, and essential vitamins and minerals, while being low in saturated fat, cholesterol, and sodium (Nikolić et al. 2026; Wu et al. 2023). It is highly adaptable and widely cultivated across diverse environments. Additionally, peas are well known for their resilience to freezing temperatures and their ability to enhance soil health through rotation with cereals, contributing to improved disease management, weed control, and nitrogen fixation (Franguelli et al. 2024; Tulbek et al. 2017).

Despite its enormous agricultural importance, pea cultivation is increasingly threatened by different stresses, particularly osmotic stress induced by drought and salinity, which severely impact growth and yield (Bagheri et al. 2023; Nadeem et al. 2019). Osmotic stress impacts plant growth by interfering with water absorption, triggering a series of physiological and biochemical alterations (Khan et al. 2022; Wahab et al. 2022). This stress is prevalent in arid and semi‐arid regions where soil salinization and drought are common (Navarro‐Torre et al. 2023). Under osmotic stress conditions, plants exhibit reduced germination rates, impaired growth, and lower productivity due to decreased water potential and ionic imbalance (Khan et al. 2022). These conditions are exacerbated by increased reactive oxygen species (ROS) production, which can overwhelm a plant's antioxidant defenses, leading to oxidative damage and stress‐induced cell death (Wu et al. 2026). In response to osmotic stress, plants have developed complex signaling pathways and stress response mechanisms. Key elements in this process are the MAPK kinases, CBL–CIPK pathway, and SNF1‐related protein kinase (SnRK) 2 family, which is crucial for osmotic stress signaling and activating stress response pathways (Chen et al. 2024). Identifying genotypes with a strong tolerance to osmotic stress in the early stage of growth is essential for breeding programs focused on enhancing crop resilience and sustainability.

Moreover, traditional breeding approaches for enhancing stress tolerance in crops often struggle due to limited genetic diversity and variable environmental factors. In contrast, in vitro techniques offer a controlled setting that isolates genotypes from external stressors like pathogens and climate variations (Sharma et al. 2024; Raoufi et al. 2021). These methods have proven effective in studying responses to various abiotic stresses, including drought, heavy metals, and salinity (Sattar et al. 2021; Miljus‐Djukic et al. 2013). Specifically, in vitro screening with osmotic stress, such as sorbitol‐induced stress, allows for precise evaluation of seed germination, growth, and development under controlled conditions (Wellpott et al. 2024; Mehmandar et al. 2023). Furthermore, indirect regeneration via callus formation provides an effective and stable platform for evaluating genotype adaptability and facilitating genetic transformation (Wang et al. 2024; Wijerathna‐Yapa and Hiti‐Bandaralage 2023).

Therefore, the study aimed to utilize in vitro screening to assess osmotic stress tolerance in a diverse collection of pea genotypes by evaluating their germination and growth responses under sorbitol‐induced stress. The primary objective was to identify highly tolerant genotypes at the early stage of growth that could enhance breeding programs and contribute to the development of resilient pea cultivars.

## 2 Materials and Methods

### 2.1 Planting Materials

The study utilized 10 varieties of commercially cultivated peas (*Pisum sativum L*.) that were selected based on their widespread cultivation throughout the country including drought and salt affected regions (Rahman et al. 2017; Azam et al. 2024) and represented seed characteristics in Table 1.

**TABLE 1:** Description of the pea genotypes and their sources used in the experiment.

Genotype name	Source/origin	Seed color	Hundred seed weight (g)
Bagha Local	Local	Cream	9.01
BARI Motor 1	Rel. var. BARI	Brown	9.84
BARI Motor 3	Rel. var. BARI	Cream	11.39
BD4142	PGRC, BARI	Brown	6.75
BD4181	PGRC, BARI	Brown	5.48
BD4209	PGRC, BARI	Brown	20.64
BD9047	PGRC, BARI	Brown	7.22
Faridpur Local	Local	Light yellow	4.93
IPSA Motor 3	BSMRAU	Gray	17.51
Sikkim Local	Nepal	Creamy white	27.38

### 2.2 Experimental Design and In Vitro Screening

The experiment was conducted at the Biotechnology Laboratory, Patuakhali Science and Technology University, Patuakhali‐8602, Bangladesh. The study was arranged in a Completely Randomized Design (CRD) with four replications and was conducted in two independent experimental runs. Each run included 40 samples in total, with 10 samples assigned per replication. Seeds were surface sterilized by rinsing in 70% (v/v) ethanol for 2 min, followed by a treatment with 50% (m/v) sodium hypochlorite (NaClO) on a rotary shaker at 50–100 rpm for 20 min. After sterilization, seeds were rinsed three times with sterile distilled water to remove any residual chemicals. Sterilized seeds (10 per replication of each variety) were then transferred into Murashige and Skoog (MS) basal medium (Murashige and Skoog 1962), which contained essential macronutrients, micronutrients, and vitamins. The medium was solidified with 0.8% (w/v) agar and supplemented with 3% (w/v) sucrose as the carbon source. Sorbitol was added at three concentrations (0.0 M, 0.1 M, and 0.2 M) to assess its impact on germination. The vials were then placed in a culture room at 24°C ± 2°C under a 16‐h photoperiod for 10–15 days, allowing seed germination and subsequent excision of explants. After 10–15 days of growth, explants were excised from the aseptically cultured seedlings. The explants included both the epicotyl and hypocotyl regions. For each replication, 10 explants were excised and transferred to a callus‐inducing medium. The callus‐inducing medium consisted of MS basal medium supplemented with 1.0 mg/L 6‐benzylaminopurine (BAP) and 2.0 mg/L naphthaleneacetic acid (NAA) for callus induction. To evaluate osmotic stress tolerance, all media formulations included varying concentrations of sorbitol (0.0 M, 0.1 M, and 0.2 M). For shoot regeneration, 10 callus explants per replication were transferred to MS basal medium supplemented with 3.0 mg/L BAP and 1.0 mg/L NAA for shoot regeneration. Media formulations again included varying sorbitol concentrations (0.0 M, 0.1 M, and 0.2 M) to assess the effects of osmotic stress on shoot regeneration. For root induction, regenerating shoots were transferred to MS medium containing 0.1 mg/L indole‐3‐acetic acid (IAA) and 0.5 mg/L indole‐3‐butyric acid (IBA). As with the previous stages, all media included varying concentrations of sorbitol (0.0 M, 0.1 M, and 0.2 M) to evaluate osmotic stress tolerance during root formation. The pH of the medium was adjusted to 5.8 using 0.1 N hydrochloric acid (HCl) or 0.1 N sodium hydroxide (NaOH), and then autoclaved at 121°C for 30 min under 15 PSI pressure. All cultures were maintained in a growth chamber at a temperature of 25°C ± 1°C, with a photoperiod of 16 h and a light intensity of 200 μmol m^−2^ s^−1^ of photosynthetic photon flux density (PPFD). Relative humidity was maintained at 60% throughout the entire culture period.

### 2.3 Growth Metrics

The growth and development of the plantlets were evaluated using the following key parameters:

#### 2.3.1 Seed Germination Rate

Seed germination was assessed 7 days after sowing. The germination rate was calculated as the percentage of seeds that successfully germinated, which was defined by the emergence of the radicle (root) from the seed coat, using the following formula:

$$\text{Seed Germination Rate}\;\left(\%\right)=\frac{\text{Number of seeds germinated}}{\text{Total number of seeds sown}}\times 100$$

#### 2.3.2 Callus Induction Percentage and Callus Diameter

Callus induction percentage and callus diameter were evaluated 4 weeks after transferring the explants to the callus‐inducing medium. The percentage of explants that developed visible callus growth was calculated by using the following formula. Callus size was measured in square millimeters (mm^2^) using a digital caliper.

$$\text{Callus Induction Percentage}=\frac{\text{Number of explants with callus}}{\text{Total number of explants cultured}}\times 100$$

#### 2.3.3 Shoot and Root Initiation Percentage, Length, and Total Fresh Weight

Shoot and root formation were recorded at 28 days of culture. The percentage of explants that developed shoots and roots was calculated using the following formulas. The length of the longest shoot and the longest root was measured in millimeters (mm), from the base to the tip. Shoot and root lengths were measured using a ruler. The fresh weight (mg) of the seedlings was determined by gently removing them from the culture medium, blotting them dry, and weighing them using a precision balance.

$$\%\text{of shoot initiation}=\frac{\text{Number of explants with shoots}}{\text{Number of explants inoculated}}\times 100$$

$$\%\text{of root initiation}=\frac{\text{Number of shoots with roots}}{\text{Number of shoots cultured}}\times 100$$

### 2.4 Statistical Analysis

A two‐way Analysis of Variance (ANOVA) was used to examine the effects of accessions and stress levels on the measured traits. Mean differences were compared using Tukey's Honestly Significant Difference (HSD) test at a 5% significance level (*p* ≤ 0.05). Heatmap clustering and the Multi‐Trait Selection Index (MTSI) were used to identify trait patterns and superior‐performing accessions. All statistical analyses and graphical visualizations were performed using R software (v4.5.3).

## 3 Results

### 3.1 Seed Germination Rate

Sorbitol‐induced osmotic stress had a significant effect (*p* ≤ 0.001) on the germination percentages of the tested pea genotypes. As the concentration of sorbitol increased, a clear decline in germination rates was observed (Figure 1 and Appendix S1). Under control conditions (0.0 M sorbitol), no significant differences were observed between the accessions. With the introduction of 0.1 M sorbitol, BD4142 demonstrated the highest germination at 63.33% followed by BARI Motor 1 and BD4209 at 56.67%, while Sikkim local exhibited the lowest germination performance at 26.67%. Under maximum stress (0.2 M sorbitol), germination percentages declined across all genotypes, with BD4142 again achieving the highest rate at 43.33% followed by BD4209 at 40% while the lowest was observed from Sikkim Local at 16.67%.

**FIGURE 1:**
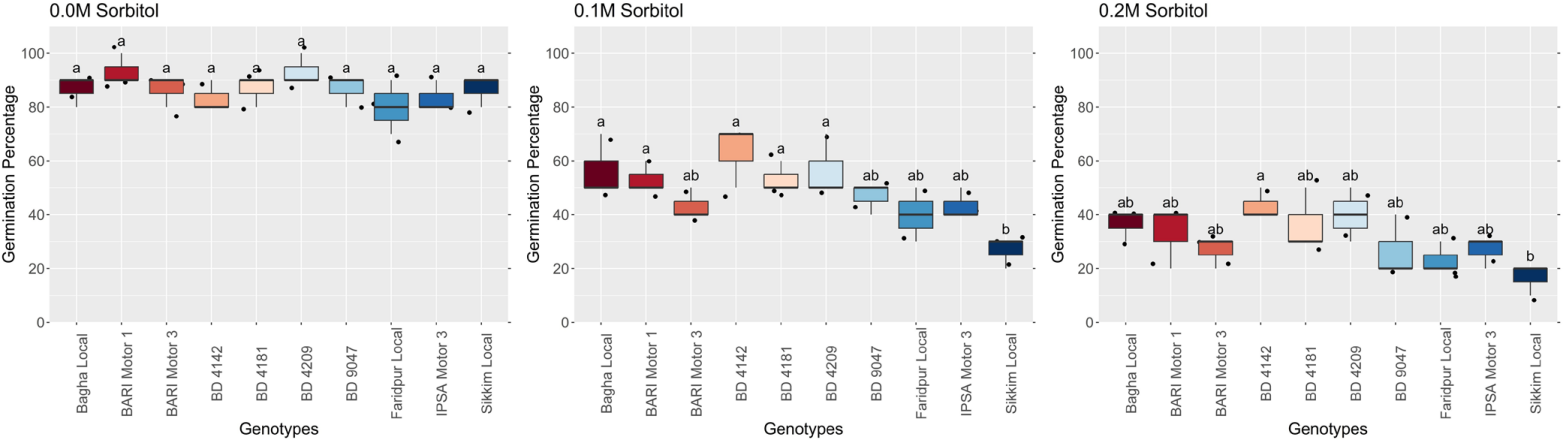
Germination rates of various pea genotypes under different levels of stress. Here, vertical bars represent the standard error.

### 3.2 Callus Formation

Callus formation exhibited significant variation (*p* ≤ 0.001) across different concentrations of sorbitol. The highest average callus formation occurred at control (0.0 M sorbitol), with a pronounced decline observed at 0.1 M and 0.2 M concentrations (Figure 2 and Appendix S2). At control, no significant differences were observed among genotypes. With increasing sorbitol concentration to 0.1 M, callus formation declined, where BD4142 still exhibited a notable response with 40% callus formation followed by BD4209 at 36.67%. At 0.2 M sorbitol, there was a further decrease in callus formation across all genotypes, where BD4142 recorded the highest callus formation at 26.67% followed by BD4209 at 23.33%, while Sikkim Local, IPSA Motor 3, and BARI Motor 3 failed to form any callus.

**FIGURE 2:**
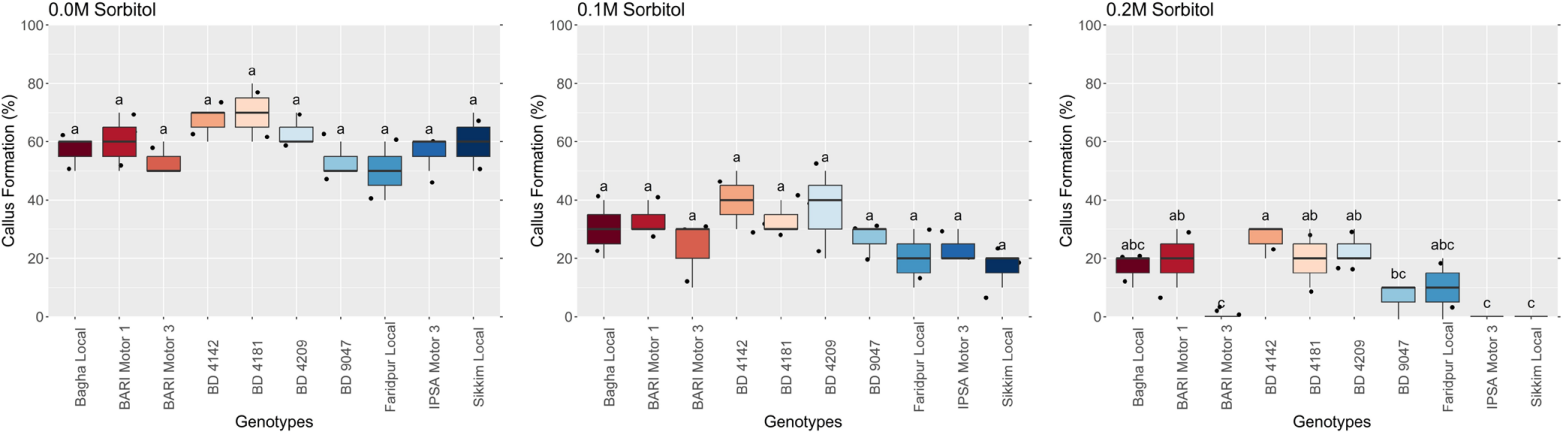
Callus formation percentage of various pea genotypes under different levels of stress. Here, vertical bars represent the standard error.

### 3.3 Size of Callus

The impact of varying sorbitol concentrations on callus size was significant (*p* ≤ 0.001). As sorbitol concentration increased from 0.0 M to 0.2 M, a marked reduction in callus size was observed in all genotypes (Figure 3 and Appendix S3). At the control (0.0 M), BD4181 exhibited the largest callus at 31.27 mm^2^, while BARI Motor 3 showed the smallest callus at 19.17 mm^2^. At 0.1 M sorbitol, the largest callus was observed in BD4142 with 22.63 mm^2^, while the smallest was in Sikkim Local with 7.23 mm^2^. At 0.2 M sorbitol, the largest callus size was again recorded from BD4142 at 15 mm^2^, whereas BD9047 produced the smallest callus at 4.63 mm^2^.

**FIGURE 3:**
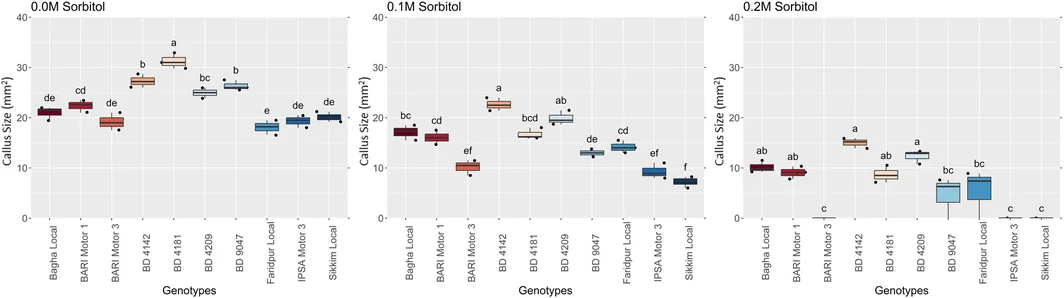
Callus size (mm^2^) of various pea genotypes under different levels of stress. Here, vertical bars represent the standard error.

### 3.4 Shoot Formation

Sorbitol concentration significantly (*p* ≤ 0.001) impacted shoot formation (Figure 4 and Appendix S4). At 0.0 M sorbitol, no significant differences were observed among the accessions. However, at 0.1 M sorbitol, BD4142 had the highest shoot formation rate at 50%, while Sikkim Local produced no shoots. At 0.2 M sorbitol, BD4142 again showed the highest shoot formation at 30%, while Sikkim Local, IPSA Motor 3, BARI Motor 3, Faridpur Local, and BD9047 failed to produce shoots.

**FIGURE 4:**
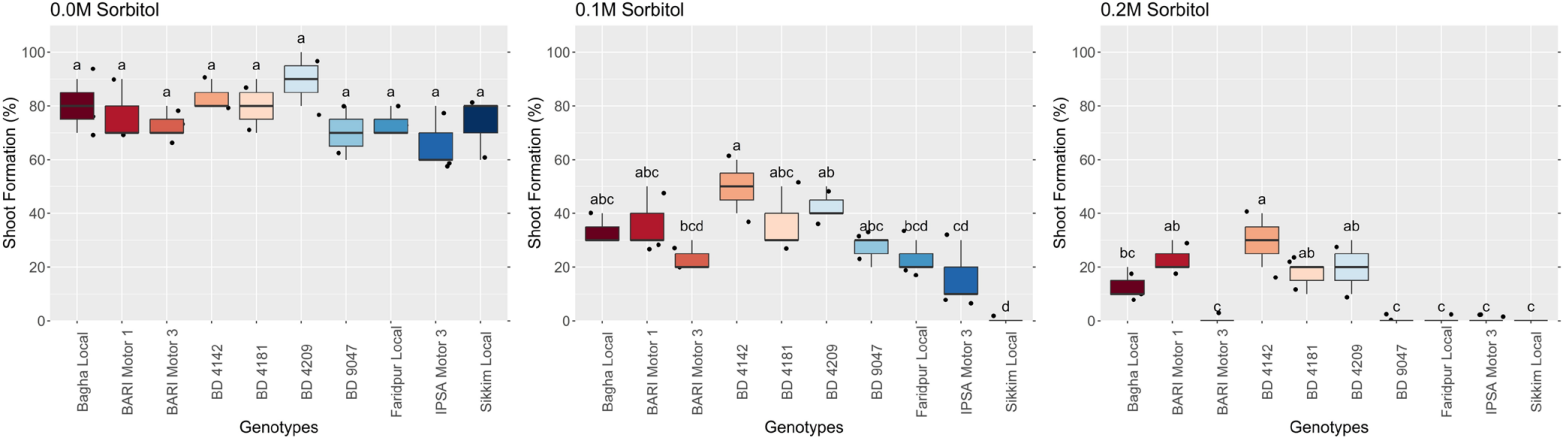
Percentage of shoot formation in various pea genotypes under different levels of stress. Here, vertical bars represent the standard error.

### 3.5 Length of Shoot

Shoot length varied significantly (*p* ≤ 0.001) across different sorbitol concentrations (Figure 5 and Appendix S5). At control (0.0 M), BD4209 exhibited the longest shoot at 85.67 mm, while IPSA Motor 3 had the shortest shoot at 68 mm. At 0.1 M sorbitol, BD4142 produced the longest shoot at 50.33 mm, while IPSA Motor 3 recorded the shortest at 18 mm. At 0.2 M sorbitol, BD4142 again showed the longest shoot at 19.33 mm, while Bagha Local had the shortest at 9.50 mm.

**FIGURE 5:**
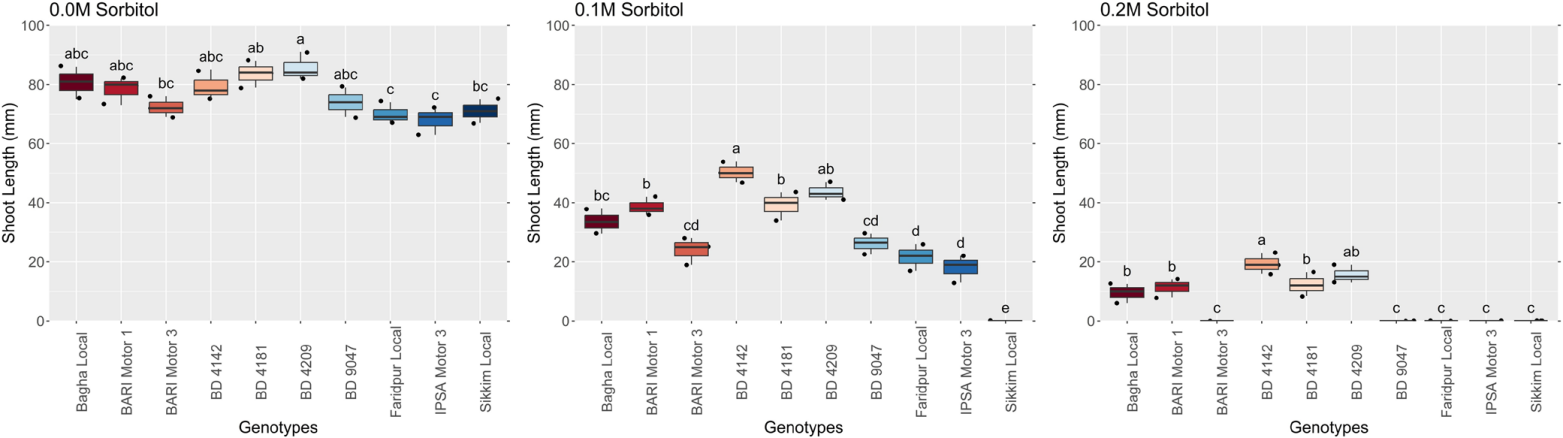
Shoot length (mm) of various pea genotypes under different levels of stress. Here, vertical bars represent the standard error.

### 3.6 Root Formation

Root formation percentages were significantly (*p* ≤ 0.001) affected by sorbitol concentration (Figure 6 and Appendix S6). At 0.0 M sorbitol, no significant differences in root formation were observed among accessions. However, with increasing sorbitol concentration, root formation decreased significantly. At 0.1 M sorbitol, BD4142 exhibited the highest root formation at 40%, followed by BD4209 at 36.67%, while Sikkim Local showed no root formation. At 0.2 M sorbitol, BD4142 again had the highest root formation rate of 23.33%, while the other accessions, including Sikkim Local, IPSA Motor 3, BARI Motor 3, Faridpur Local, and BD9047, failed to produce roots.

**FIGURE 6:**
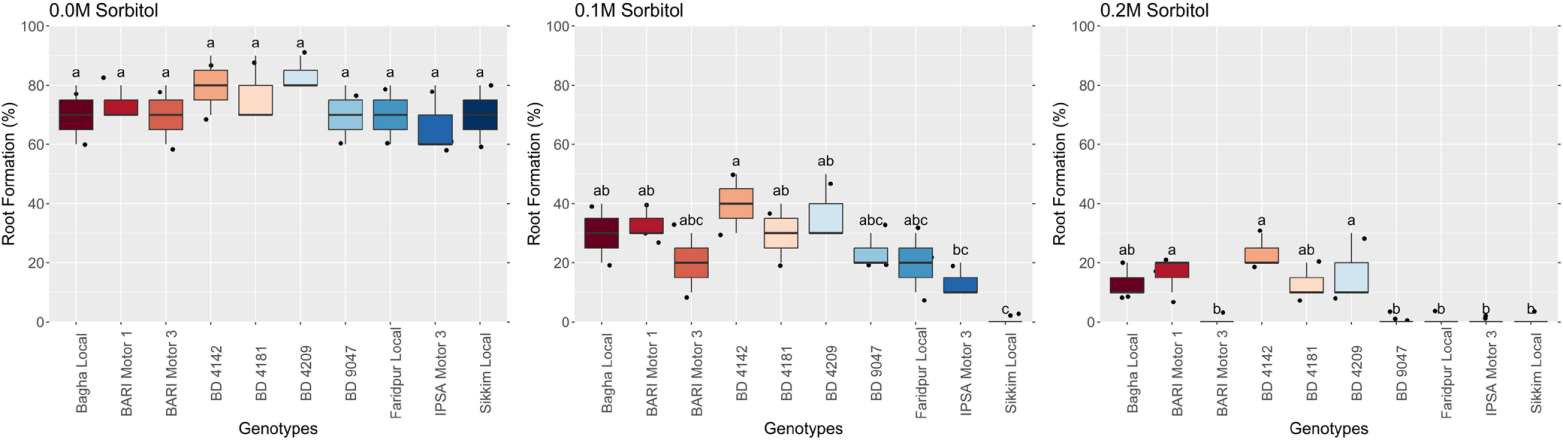
Percentage of root formation in various pea genotypes under different levels of stress. Here, vertical bars represent the standard error.

### 3.7 Length of the Longest Root

Root length exhibited significant variation (*p* ≤ 0.001) among different concentrations of sorbitol (Figure 7 and Appendix S7). At 0.0 M sorbitol, Bagha Local exhibited the longest root length at 124 mm, similar to BD4181 and BD4142, while IPSA Motor 3 had the shortest root at 84.67 mm. At 0.1 M sorbitol, BD4142 produced the longest root at 76.67 mm, followed by BD4209 at 68 mm, while IPSA Motor 3 had the shortest root at 31 mm. At 0.2 M sorbitol, BD4142 again had the longest root at 32.67 mm, while Bagha Local produced the shortest root at 18.50 mm.

**FIGURE 7:**
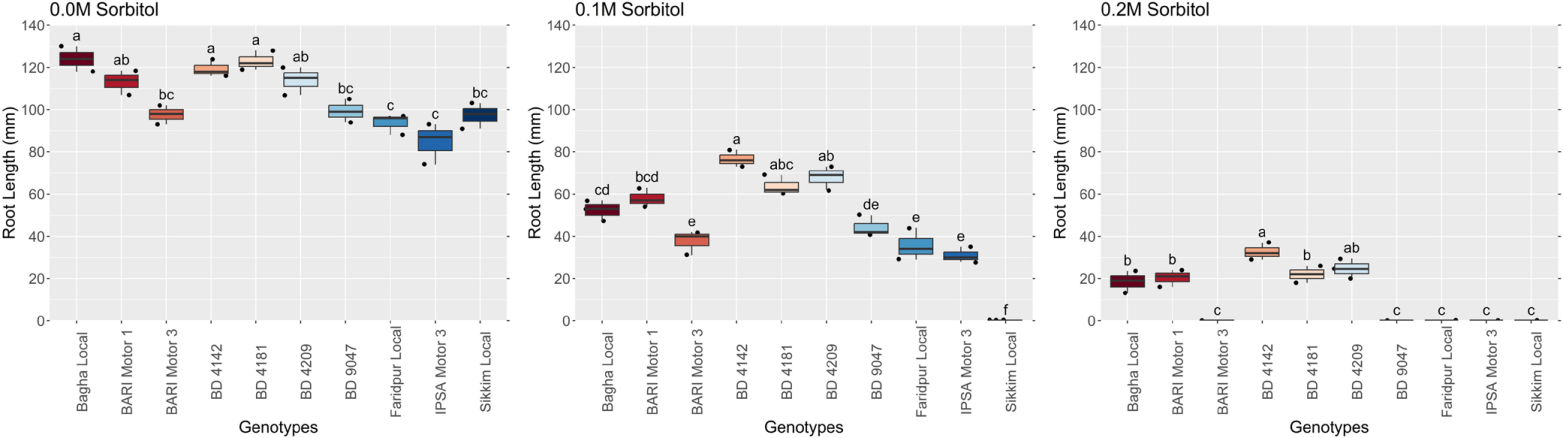
Root length (mm) of various pea genotypes under different levels of stress. Here, vertical bars represent the standard error.

### 3.8 Plant Fresh Weight

Plant fresh weight varied significantly (*p* ≤ 0.001) across different sorbitol concentrations (Figure 8 and Appendix S8). At 0.0 M sorbitol, BD4181 had the highest fresh weight at 1095 mg, followed by Bagha Local at 1023.33 mg, BD4209 at 1011 mg, and BD4142 at 1005 mg. In contrast, IPSA Motor 3 recorded the lowest fresh weight at 776.62 mg. At 0.1 M sorbitol, fresh weight generally decreased for most varieties. BD4142 maintained the highest fresh weight at 555 mg, followed by BD4209 at 493.30 mg and BD4181 at 446.58 mg while Sikkim Local exhibited no measurable fresh produce at this concentration. At 0.2 M sorbitol, the reduction in fresh weight was more pronounced. BD4142 still had the highest fresh weight at 258.32 mg, followed by BD4209 at 201.72 mg, whereas BARI Motor 3, BD9047, Faridpur Local, IPSA Motor Pea 3, and Sikkim Local failed to produce any measurable plant parts.

**FIGURE 8:**
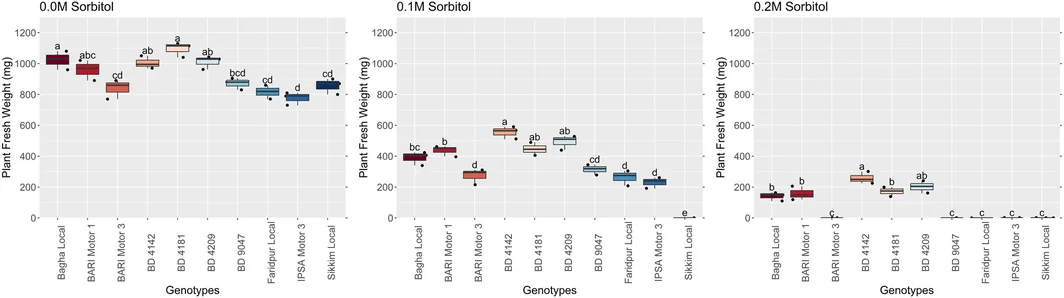
Plant fresh weight (mg) of various pea genotypes under different levels of stress. Here, vertical bars represent the standard error.

### 3.9 Two‐Way ANOVA

The two‐factor analysis of variance supported these findings, revealing that the concentration of sorbitol in the culture medium had the most significant impact on all measured traits, followed by genotype and the interaction between genotype and sorbitol concentration, both of which demonstrated similar effect sizes (Table 2).

**TABLE 2:** Two‐way analysis of variance (ANOVA) showing mean squares for the effects of genotype and sorbitol concentration on germination and growth traits.

Source of variation	Germination rate	Callus formation	Size of callus	Shoot formation	Length of shoot	Root formation	Length of root	Plant fresh weight
Genotype	7.04***	8.70***	62.76***	15.15***	54.24***	9.64***	102.70***	73.34***
Sorbitol	411.14***	296.40***	730.95***	563.82***	2804.91***	532.32***	3371.03***	3357.01***
Genotype × Sorbitol	1.50	1.01	6.93***	1.80*	7.55***	1.22	11.00***	7.09***
Error	59.2	56.9	2.78	62	13	64	20	1633

*Note: p* ≤ 0.1. NS indicates a non‐significant effect.

* *p* ≤ 0.05.

** *p* ≤ 0.01.

*** *p* ≤ 0.001.

### 3.10 Heatmap Cluster Analysis

Cluster analysis classified the genotypes into four distinct groups based on their sensitivity to sorbitol‐induced stress, utilizing all the measured traits (Figure 9). The first cluster identified BD4142 and BD4209 as tolerant to the stress. The second cluster comprised BD4181, BARI Motor 1, and Bagha Local, which were categorized as having moderate tolerance. The third cluster consisted of Sikkim Local, which was classified as highly susceptible. The fourth cluster included four genotypes with lower osmotic tolerance: IPSA Motor 3, BARI Motor 3, Faridpur Local, and BD9047.

**FIGURE 9:**
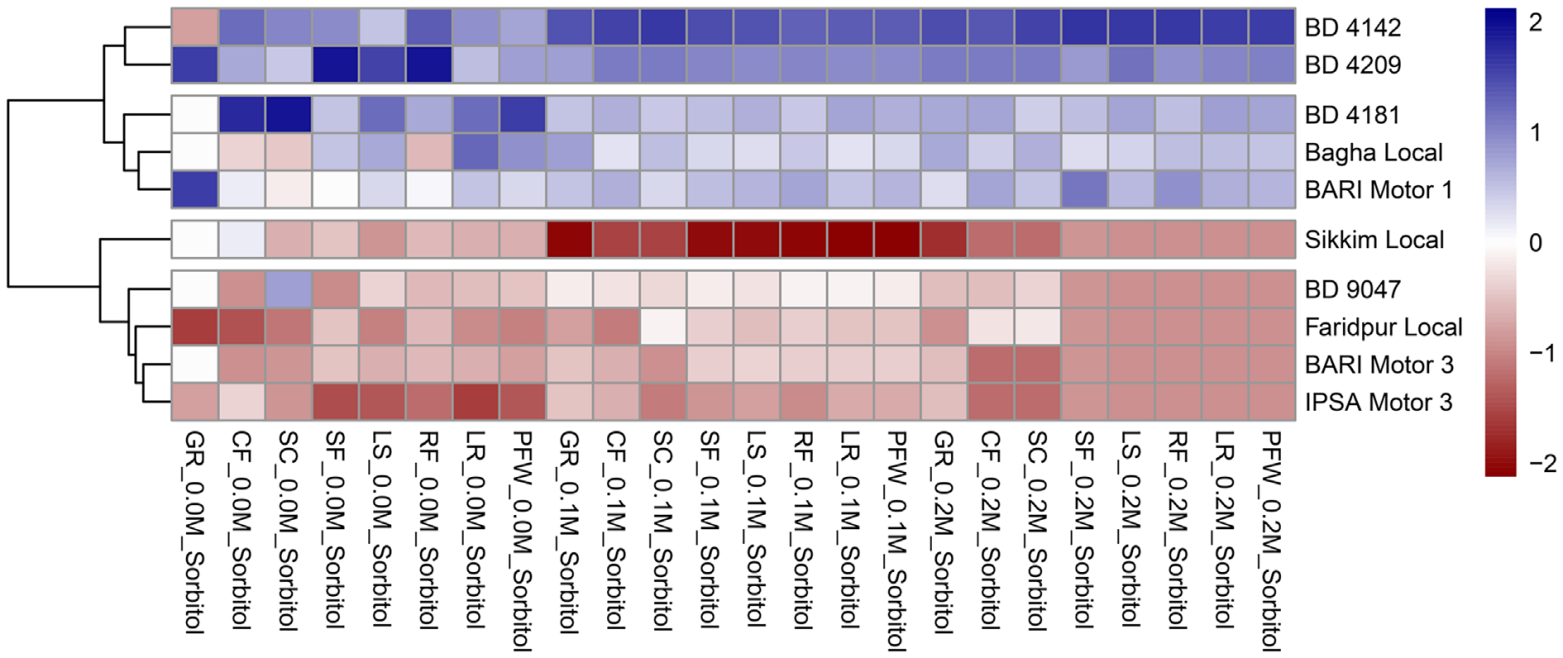
Heatmap based on the response of various pea genotypes under sorbitol‐induced stress. Here, GR = Germination Rate (%), CF = Callus Formation (%), SC = Size of Callus (mm^2^), SF = Shoot Formation (%), LS = Length of Shoot (mm), RF = Root Formation (%), LR = Length of Root (mm), PFW = Plant Fresh Weight (mg).

### 3.11 Multi‐Trait Selection Index (MTSI)

The MTSI analysis evaluated pea genotypes according to their desired trait values (Figure 10). The red circle indicates the threshold for selecting stable genotypes (Olivoto and Lúcio 2020). Among them, BD4142 and BD4209 were identified as the most stable compared to the others.

**FIGURE 10:**
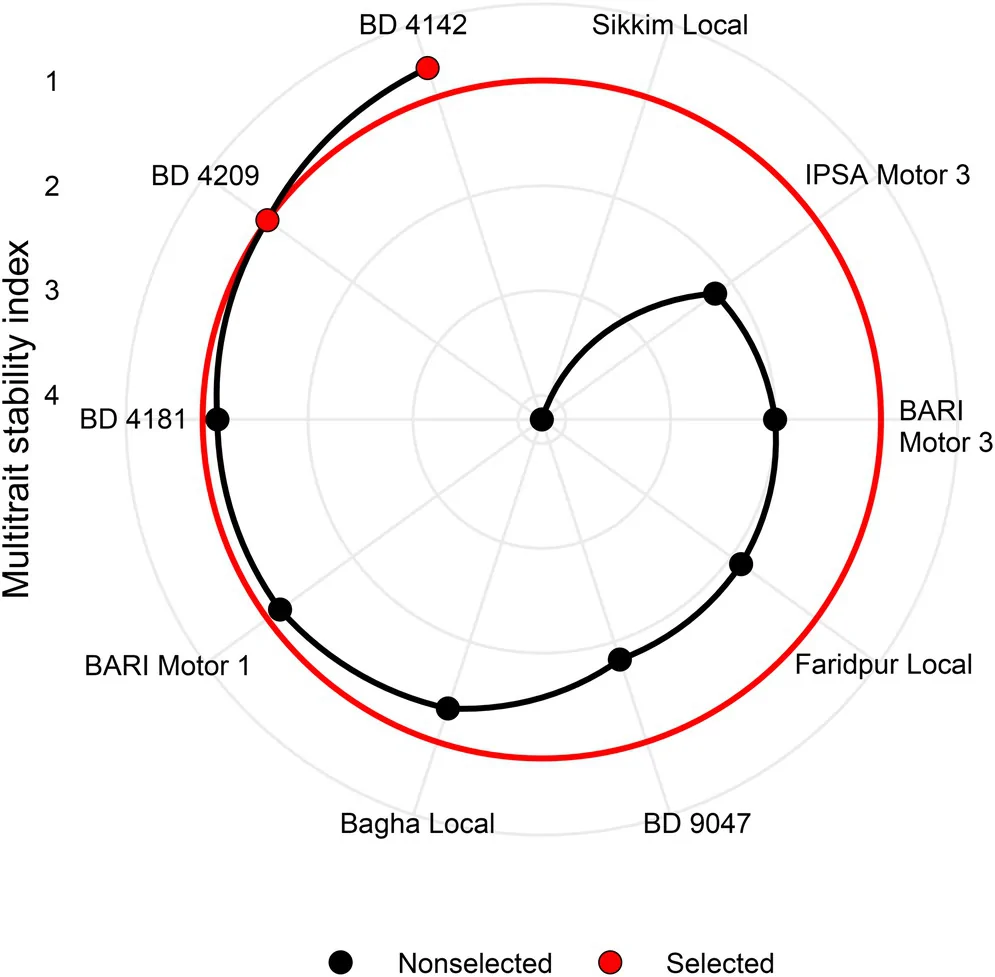
Ranking of various pea genotypes based on Multi‐Trait Selection Index (MTSI).

## 4 Discussion

The findings of this study underscore the significant effects of sorbitol‐induced osmotic stress on various growth responses in pea ( *Pisum sativum* ) genotypes. These processes include germination, callus formation, shoot and root development, and plant fresh weight. The results provide valuable insights into the genotypic variability in response to osmotic stress, which is essential for developing stress‐tolerant cultivars.

Germination is a critical stage in the plant life cycle, and it is highly sensitive to environmental stresses (Zhang et al. 2025). In this study, the negative impact of osmotic stress, as induced by increasing concentrations of sorbitol (0.0 M to 0.2 M), was evident through declining germination rates. This finding aligns with previous reports by Zhang et al. (2010) and Jain et al. (2010) that demonstrated a similar trend of reduced germination percentages under osmotic stress. At the highest sorbitol concentration (0.2 M), germination rates varied between 16.67% and 43.33%, highlighting the detrimental effect of osmotic stress on seed water uptake and enzyme activity essential for germination (Khosravi et al. 2022). Among the genotypes tested, BD4142 exhibited the highest germination rates, followed by BD4209, suggesting that these genotypes have a higher tolerance to osmotic stress. Conversely, Sikkim Local showed poor germination under stress, which indicates limited adaptability to osmotic challenges.

Callus formation is another key indicator of plant regenerative capacity, and the results revealed a significant decline in callus formation as sorbitol concentration increased. Sorbitol's role as an osmotic agent likely inhibited callus growth by reducing water potential, thereby restricting nutrient and water availability to the tissue culture (Zhao et al. 2021). BD4142 demonstrated superior callus formation even under higher sorbitol concentrations, followed by BD4209, further emphasizing their robustness in tissue culture applications. However, the failure of BARI Motor 3, Sikkim Local, and IPSA Motor 3 to produce callus at the highest sorbitol concentration suggests a limitation in their regenerative potential, which could hinder their application in regeneration protocols.

The effects of sorbitol‐induced osmotic stress were also evident in plant development, with significant reductions in shoot formation, root formation, shoot length, root length, and plant fresh weight across all genotypes as sorbitol levels increased. This finding is consistent with studies by Pacyga et al. (2024) and Zhao et al. (2021), which showed that osmotic stress adversely impacts plant growth by lowering water potential and inhibiting water uptake. Notably, BD4142 and BD4209 exhibited superior performance in terms of shoot and root formation, with BD4142 showing the highest shoot and root length under 0.2 M sorbitol. These results suggest that these genotypes possess mechanisms that allow them to adapt to osmotic stress, such as enhanced root growth, which is commonly observed in drought and salinity stress responses as a strategy to improve water uptake Karmaker et al. (2024). Similar reductions in shoot and root length with increasing sorbitol concentrations have been documented in *Salvadora persica* Bundig et al. (2017).

The decline in plant fresh weight and other growth parameters under increasing osmotic stress may be associated with elevated sorbitol levels, which can disrupt cell division, cytoplasmic volume, turgor maintenance, and nutrient balance by limiting water availability and uptake. Prolonged osmotic stress may also impair nutrient acquisition and induce ionic toxicity, further restricting plant growth (Wu et al. 2026; Yadav et al. 2022; Ashraf 2005). These factors can ultimately reduce the biosynthesis of critical growth‐related enzymes and hormones, thereby hindering the plant's ability to grow and develop under osmotic stress (Bor et al. 2003). The differential responses of pea genotypes to osmotic stress are consistent with the earlier findings by Wishart et al. (2014).

The differential responses of pea genotypes to osmotic stress were further corroborated by the cluster analysis, which successfully categorized the genotypes based on their sensitivity to osmotic stress. BD4142 and BD4209 emerged as the most tolerant genotypes, while Sikkim Local was identified as highly susceptible. The Multi‐Trait Selection Index (MTSI) also identified BD4142 as the most stable genotype, reinforcing its suitability for breeding programs focused on enhancing stress tolerance.

## 5 Conclusion

The study successfully identified and classified different pea ( *Pisum sativum* L.) genotypes based on their resistance to osmotic stress induced by varying concentrations of sorbitol in in vitro systems. The results demonstrated significant declines in germination, callus formation, and growth metrics such as shoot length, root length, and plant fresh weight with increasing sorbitol concentration. Notably, BD4142 emerged as the most resilient genotype, exhibiting the highest germination rates and growth under the maximum sorbitol concentration, while Sikkim Local was identified as the most susceptible. Further field trials of these genotypes are recommended considering their biochemical and physiological responses to confirm their suitability as osmotic stress tolerant pea cultivars.

## Funding

The authors have nothing to report.

## Conflicts of Interest

The authors declare no conflicts of interest.

## Supporting information


**Appendix S1:** ANOVA for Germination (%).
**Appendix S2:** ANOVA for Callus Formation (%).
**Appendix S3:** ANOVA for Size of Callus (mm).
**Appendix S4:** ANOVA for Shoot Formation (%).
**Appendix S5:** ANOVA for Length of shoot (mm).
**Appendix S6:** ANOVA for Root formation (%).
**Appendix S7:** ANOVA for Length of root (mm).
**Appendix S8:** ANOVA for Plant fresh weight (mg).

## Data Availability

The data that supports the findings of this study are available in the Supporting Information of this article.
