# Supplementary material for: Screening of Different Pea ( Pisum sativum L.) Genotypes for Osmotic Stress Resistance Using Selective in Vitro Systems
Source: Plant Environ Interact. 2026 Sep 29;7(5):e70215. doi: 10.1002/pei3.70215 (PMC13621232; doi:10.1002/pei3.70215)
Supplement: Supplementary file 1 [file PEI3-7-e70215-s001.docx]

```
Appendices:

Supplementary Appendix 1: ANOVA for Germination (%)
                             Df  Sum Sq Mean Sq  F value    Pr(>F)
data$Replications             1   166.7   166.7   3.0412   0.08638 .
data$Treatments               2 26975.6 13487.8 246.1172 < 2.2e-16 ***
data$Variety                  9  6071.1   674.6  12.3091 1.062e-10 ***
data$Treatments:data$Variety 18  1602.2    89.0   1.6242   0.08325 .
Residuals                    59  3233.3    54.8
---
Signif. codes:  0 â€˜***â€™ 0.001 â€˜**â€™ 0.01 â€˜*â€™ 0.05 â€˜.â€™ 0.1 â€˜ â€™ 1

Supplementary Appendix 2: ANOVA for Callus Formation (%)
                             Df  Sum Sq Mean Sq  F value    Pr(>F)
data$Replications             1   106.7   106.7   1.8366    0.1805
data$Treatments               2 25220.0 12610.0 217.1177 < 2.2e-16 ***
data$Variety                  9 11601.1  1289.0  22.1941 7.439e-16 ***
data$Treatments:data$Variety 18   935.6    52.0   0.8949    0.5865
Residuals                    59  3426.7    58.1
---
Signif. codes:  0 â€˜***â€™ 0.001 â€˜**â€™ 0.01 â€˜*â€™ 0.05 â€˜.â€™ 0.1 â€˜ â€™ 1

Supplementary Appendix 3: ANOVA for Size of Callus (mm)
                             Df Sum Sq Mean Sq  F value    Pr(>F)
data$Replications             1    0.5    0.52   0.2212    0.6398
data$Treatments               2 3958.8 1979.40 837.8608 < 2.2e-16 ***
data$Variety                  9 1744.6  193.85  82.0542 < 2.2e-16 ***
data$Treatments:data$Variety 18  359.8   19.99   8.4620  1.59e-10 ***
Residuals                    59  139.4    2.36
---
Signif. codes:  0 â€˜***â€™ 0.001 â€˜**â€™ 0.01 â€˜*â€™ 0.05 â€˜.â€™ 0.1 â€˜ â€™ 1

Supplementary Appendix 4: ANOVA for Shoot Formation (%)
                             Df Sum Sq Mean Sq  F value  Pr(>F)
data$Replications             1    107   106.7   2.2264 0.14100
data$Treatments               2  53247 26623.3 555.6993 < 2e-16 ***
data$Variety                  9  14046  1560.6  32.5742 < 2e-16 ***
data$Treatments:data$Variety 18   1864   103.6   2.1620 0.01384 *
Residuals                    59   2827    47.9
---
Signif. codes:  0 â€˜***â€™ 0.001 â€˜**â€™ 0.01 â€˜*â€™ 0.05 â€˜.â€™ 0.1 â€˜ â€™ 1


Supplementary Appendix 5: ANOVA for Length of shoot (mm)
                             Df Sum Sq Mean Sq   F value    Pr(>F)
data$Replications             1     38      38    4.5531   0.03703 *
data$Treatments               2  70909   35455 4203.8333 < 2.2e-16 ***
data$Variety                  9  10461    1162  137.8130 < 2.2e-16 ***
data$Treatments:data$Variety 18   2066     115   13.6072 8.221e-15 ***
Residuals                    59    498       8
---
Signif. codes:  0 â€˜***â€™ 0.001 â€˜**â€™ 0.01 â€˜*â€™ 0.05 â€˜.â€™ 0.1 â€˜ â€™ 1


Supplementary Appendix 6: ANOVA for Root formation (%)
                             Df Sum Sq Mean Sq  F value    Pr(>F)
data$Replications             1     15    15.0   0.3610  0.550264
data$Treatments               2  39620 19810.0 476.7328 < 2.2e-16 ***
data$Variety                  9  10512  1168.0  28.1088 < 2.2e-16 ***
data$Treatments:data$Variety 18   1691    94.0   2.2609  0.009842 **
Residuals                    59   2452    41.6
---
Signif. codes:  0 â€˜***â€™ 0.001 â€˜**â€™ 0.01 â€˜*â€™ 0.05 â€˜.â€™ 0.1 â€˜ â€™ 1

Supplementary Appendix 7: ANOVA for Length of root (mm)
                             Df Sum Sq Mean Sq   F value    Pr(>F)
data$Replications             1     79      79    7.6354  0.007622 **
data$Treatments               2 129356   64678 6223.6062 < 2.2e-16 ***
data$Variety                  9  23804    2645  254.5005 < 2.2e-16 ***
data$Treatments:data$Variety 18   4950     275   26.4628 < 2.2e-16 ***
Residuals                    59    613      10
---
Signif. codes:  0 â€˜***â€™ 0.001 â€˜**â€™ 0.01 â€˜*â€™ 0.05 â€˜.â€™ 0.1 â€˜ â€™ 1


Supplementary Appendix 8: ANOVA for Plant fresh weight (mg)
                             Df   Sum Sq Mean Sq   F value Pr(>F)
data$Replications             1       60      60    0.0941 0.7602
data$Treatments               2 10446331 5223165 8243.2325 <2e-16 ***
data$Variety                  9  1363033  151448  239.0163 <2e-16 ***
data$Treatments:data$Variety 18   290389   16133   25.4607 <2e-16 ***
Residuals                    59    37384     634
---
Signif. codes:  0 â€˜***â€™ 0.001 â€˜**â€™ 0.01 â€˜*â€™ 0.05 â€˜.â€™ 0.1 â€˜ â€™ 1
```
